# Role of an e-Health Intervention in Holistic Healthcare: A Quasiexperiment in Patients Undergoing Cardiac Catheterization in Taiwan

**DOI:** 10.1155/2021/6692952

**Published:** 2021-03-17

**Authors:** Jian-Rong Peng, Hung-Chi Su, Chia-Pin Lin, Chun-Chi Chen, Chi-Jen Chang, Siou-Ling Gong, Pao-Hsien Chu

**Affiliations:** ^1^Department of Cardiology, New Taipei Municipal TuCheng Hospital, Chang Gung Memorial Hospital, New Taipei, Taiwan; ^2^Department of Cardiology, Chang Gung Memorial Hospital, Linkou Medical Center, Taoyuan, Taiwan; ^3^College of Medicine, Chang Gung University, Taoyuan, Taiwan

## Abstract

**Background:**

The use of electronic health (e-health) resources is emerging as an alternative method to improve the secondary prevention of coronary artery disease (CAD). The aim of this study was to describe the influence of an e-health application in holistic healthcare for patients with CAD.

**Methods:**

A quasiexperiment with nonequivalent groups design recruited outpatients with a high risk of CAD admitted for cardiac catheterization. They were divided into two groups. Before the procedure, the control group received traditional patient education, and the intervention group watched videos on Internet-based social media. EQ-5D and FACIT–Sp-12 questionnaires were used as outcome measures of interest, and they were administered before and after the procedure and at the first return visit to the outpatient clinic after discharge. The effect of each intervention was tested using a linear mixed effects model. In addition, the 90-day readmission rate was also studied.

**Results:**

A total of 300 patients were divided into intervention and control groups (150 patients in each group). The interaction effect of EQ-5D was not statistically significant; however, improvements in FACIT–Sp-12 were greater in the intervention group from baseline to before discharge (regression coefficient (*B*) = 1.70, *p* < 0.001) and from baseline to postdischarge first outpatient visit (*B* = 1.81, *p* < 0.001). Moreover, the 90-day readmission rate was significantly lower in the intervention group (14% vs. 18.7%; *p*=0.016, log-rank test).

**Conclusions:**

e-health intervention with easily accessible Internet-based social media is a promising model to meet the holistic needs of patients with CAD in the modern era.

## 1. Introduction

Electronic health (e-health) refers to health services and information delivered or enhanced through the Internet and related technologies [1]. Mobile health (m-health), a subset of e-health, means the use of mobile computing and communication technologies in healthcare and public health [2]. As most mobile devices can access the Internet, e-health is widely applied to the general public, including electronic medical records, personal health records, electronic communication between patients and providers, and education programs [3]. The European Society of Cardiology also recommends the use of e-health resources to support remote clinical care and improve psychosocial health in patients with cardiovascular diseases (CVDs) [4–6].

CVDs are the leading causes of deaths worldwide, accounting for 30% of all deaths. Of these deaths, 50% is due to heart attack, and ischemic heart disease causes the most deaths worldwide [7, 8]. For patients with suspected coronary artery disease (CAD), accurate diagnostic assessment with invasive coronary angiography and cardiac catheterization (CC) is necessary to assess the prognosis and guide the choice of revascularization procedure such as percutaneous coronary intervention (PCI) or coronary artery bypass graft surgery (CABG) [9, 10].

With advances in material science and medical technology, CC has become a very common diagnostic procedure for patients with suspected CAD. As an invasive procedure, CC can cause significant patient anxiety [11], which may have negative implications on healthcare perception, expectations, decision-making, quality of life [12–15], and clinical outcomes [16]. To reduce patient anxiety and improve patient satisfaction, holistic healthcare can be used that considers not only physical health but also spiritual well-being [17].

In Taiwan, the average length of a scheduled hospital stay for CC and/or PCI is three days from admission to discharge. Within such a short period, it is not easy to provide adequate information to the patients. Therefore, a more effective informational education model is needed to provide holistic healthcare. The purpose of this study was to describe the influence of an e-health application in patients with suspected or established CAD undergoing CC.

## 2. Patients and Methods

### 2.1. Study Population

**Figure 1:**
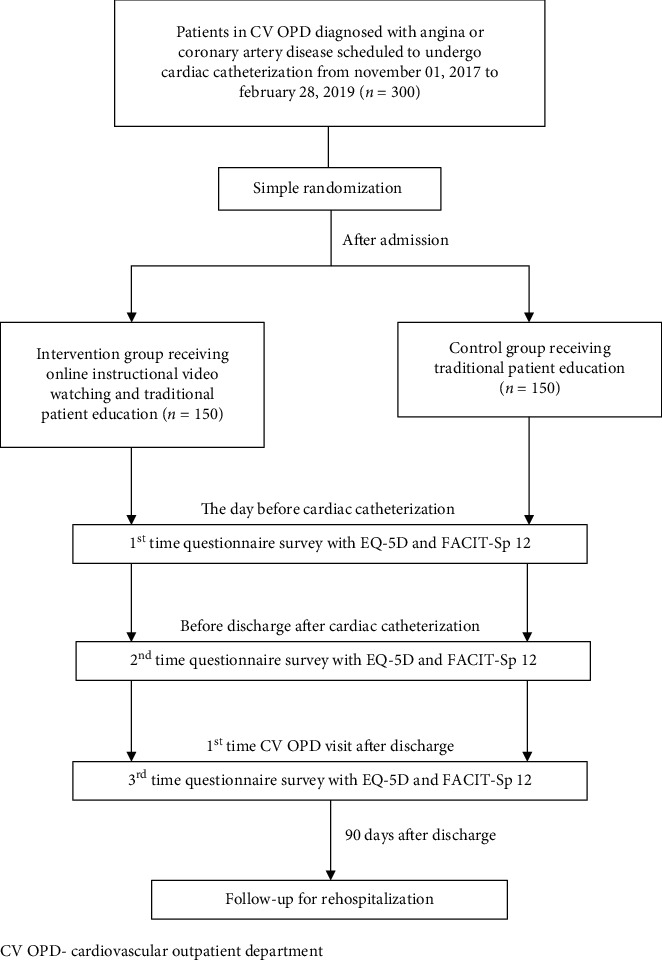
Flow chart of the study.

This study was a quasiexperimental research with a two-nonequivalent group prospective comparison design [18]. Outpatients from our cardiovascular outpatient department (CV OPD) with suspected or established CAD who were scheduled to undergo elective CC were assigned to two groups: the control group and intervention group (Figure 1). The patients in the control group received traditional ward-based patient education, including informed consent for CC by physicians and standard education and handouts on CC by clinical nurses before the procedure. In addition to the traditional patient education, those in the intervention group watched an instructional video produced by cardiologists and nurses on Internet-based social media. The length of the video was 11 minutes and included four major parts: (1) introduction to CAD, (2) indications for CC and PCI, (3) precautions after CC or PCI, and (4) essentials of primary or secondary prevention of CAD. The patients were given the link to the social media website, and after searching for the video using keywords, they could watch it online as many times as they wanted on their own mobile devices such as laptops, tablets, or smartphones. To avoid intergroup interference, e.g., the patients in the control group also watched the instructional video online, and we recruited the control group before the intervention group in different periods (Figure 1).

### 2.2. Instruments

The two instruments used as the indices of holistic healthcare in this study were the EQ-5D and the Functional Assessment of Chronic Illness Therapy–Spiritual Well-Being Scale (FACIT–Sp-12) questionnaires, which measure the respondents' self-reported health status and spiritual well-being, respectively.

#### 2.2.1. EQ-5D-5L

The five-level of ED-5Q version(EQ-5D-5L) is a standardized instrument used to measure generic health status, also involving cardiovascular disease [19, 20], and is composed of two parts: EQ-5D-5L descriptive system and EQ visual analogue scale (EQ-VAS), both measures self-perceived heath status [21, 22]. The EQ-5D-5L is a five-dimension-specific rating scale including a 5-point scale ranging from 1 to 5 according to the severity in each case (mobility, self-care, usual activities, pain/discomfort, and anxiety/depression). A lower total score indicates better subjective health status or quality of life. In contrast, the EQ-VAS is rated on a scale from 0 to 100, indicating the worst to the best imaginable health status.

#### 2.2.2. FACIT–Sp-12

The FACIT–Sp-12 is a self-administered questionnaire supporting three or two factors (peace/meaning and faith). It contains 12 items (eight for peace/meaning, four for faith) with a 4-point Likert scale that measures spiritual well-being in people with chronic illnesses [23, 24]. Every subscale measures different aspects of spiritual well-being: peace for a sense of harmony and peace deriving from a connection deriving from something larger than one's self, mean for a sense of purpose and significance from a connection to something larger than one's self, and faith for a sense of strength and comfort from one's faith and spiritual beliefs [25]. A higher score indicates better spiritual well-being.

### 2.3. Study Design

The questionnaires were given three times in both groups. First, after the ward-based education and/or watching the online video and before CC. Second, after the procedure but prior to discharge. Third, at the first return visit to the CV OPD after discharge. Apart from the questionnaires, a 90-day follow-up visit after discharge was arranged. By analyzing the questionnaire results and outcomes of postdischarge follow-up between the two groups, the influence of watching the online video before the procedure was investigated.

### 2.4. Statistics

Baseline characteristics of the intervention and control groups were compared using the independent sample test for continuous variable and the chi-square test for categorical variable. EQ-5D and FACIT–Sp-12 scores were compared between the two groups at each of the three measurements (before catheterization, before discharge, and postdischarge first outpatient visit) using the independent sample test. The readmission rate during 90 days of follow-up was compared between the two groups using the log-rank test. The effect of the intervention was assessed using a linear mixed effects model which included main effects of intercept, covariates, the study group (1 = intervention; 0 = control) and measurement (three time points), and two-way interactions of “group × measurement.” There were three random effects: the intercept, the slope of time, and the residual. The selected covariates were age, sex, smoking, coronary artery bypass graft, length of hospitalization, and use of nitrates. The effect of the intervention was confirmed if the two-way interaction effect was significant. All tests were two-tailed, and *p* value < 0.05 was considered to be statistically significant. No adjustments for multiple testing (multiplicity) were made in this study. Data analyses were conducted using SPSS version 25 (IBM SPSS Inc., Chicago, Illinois). The analytical results are summarized in Table S1.

## 3. Results

### 3.1. Baseline Characteristics

From November 1, 2017 through February 28, 2019, a total of 300 patients scheduled to undergo CC were randomly assigned to the intervention and control groups (150 patients in each group). There were no significant differences in baseline characteristics between the two groups except for age (*p*=0.019) and length of hospital stay (*p*=0.004). The patients in the intervention group were younger (62.85 years) than those in the control group (65.56 years), and the length of hospitalization was shorter in the intervention group (3.54 days) than in the control group (4.89 days) (Table 1).

### 3.2. Outcome Measures at Each Measurement

Patients in the intervention group reported significantly lower mean EQ-5D-5L scores than the control group at all three time points (*p*=0.014, <0.001, and <0.001, respectively). There was no obvious difference in EQ-VAS between the two groups. In terms of spiritual well-being, the FACIT–Sp-12 score in the intervention group was significantly higher than that in the control group at the first return visit to the CV OPD (*p*=0.016). In addition, the intervention group had better spiritual well-being with regards to faith before discharge (*p* < 0.001) and at first return to outpatient clinic than the control group (*p* < 0.001) (Table 2).

### 3.3. Readmission Rate

**Figure 2:**
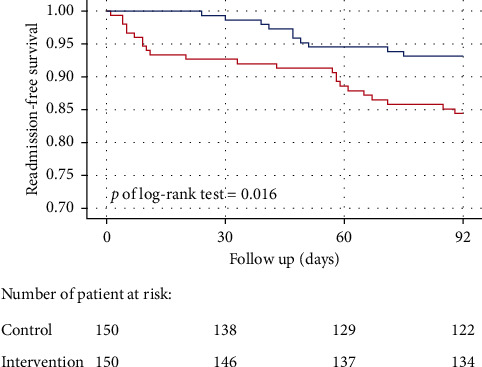
Kaplan–Meier survival curves of 90-day readmission of patients in the control and intervention groups. The readmission rate within 90 days after discharge was significantly lower in the intervention group (red line) compared with the control (blue line).

With respect to the postdischarge follow-up at 90 days, the overall readmission rate of patients in the control group was significantly higher than that in the intervention group (18.7% vs. 14%; *p*=0.016, log-rank test; Figure 2). By analyzing the etiologies of readmission, patients in the control group were more likely to be readmitted within the first 3 months after the index hospitalization due to heart failure decompensation or other comorbidities such as diabetes mellitus, chronic kidney disease, or peripheral artery disease (Table 3).

### 3.4. Intervention Effect on Outcome Measures

**Figure 3:**
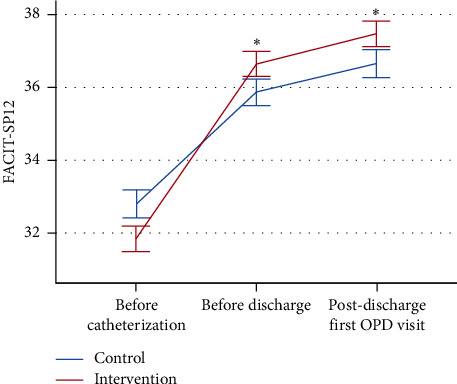
The means and standard errors of FACIT–SP12 of the intervention and control groups at each measurement. The improvement of the total FACIT–Sp-12 score from baseline was significantly more in the intervention group before discharge and in the first postdischarge visit to OPD. FACIT–SP, the Functional Assessment of Chronic Illness Therapy–Spiritual Well-Being Scale; ^*∗*^indicates that the improvement from baseline to follow-up was significantly different between the two groups (*p* < 0.05).

After controlling the selected covariates, including age, sex, smoking, CABG, length of hospitalization, and use of nitrates, the linear mixed effects model demonstrated that the interaction effects of EQ-5D-5L and EQ-VAS between the two groups were not statistically significant, suggesting that the improvement in outcome measures was not superior in the intervention group. However, the improvement in FACIT–Sp-12 was greater in the intervention group than in the control group from baseline to before discharge (regression coefficient (*B*) = 1.70, *p* < 0.001) and from baseline to postdischarge first CV OPD visit (*B* = 1.81, *p* < 0.001) (Figure 3).

## 4. Discussion

### 4.1. Effects of the e-Health Intervention

In this quasiexperimental prospective study involving cardiac outpatients scheduled to undergo CC, the preprocedural intervention of watching an online instructional video could more effectively improve the quality of holistic healthcare before discharge (after CC) and in postdischarge follow-up compared to traditional ward-based patient education.

Despite the improvement of subjective health status in EQ-5D not superior to the control, the patients in the intervention group indeed had significantly lower score in EQ-5D-5L before and after CC and in the first postdischarge visit to OPD. The EQ-5D-5L is widely used to assess general health status and evaluate four physical dimensions and one mental or spiritual dimension about anxiety/depression [21]. Because all the participants in this study were recruited from OPD, their physical condition should have been relatively stable. Accordingly, differences in EQ-5D-5L would result from the mental or spiritual dimension, suggesting less anxiety/depression the intervention group had at all the three timepoints.

The improvement of FACIT–Sp-12 from baseline to predischarge and first postdischarge OPD visit also corresponded to higher spiritual well-being in the intervention group. Interestingly, the intervention group had significantly lower faith subscale scores than the control before CC. It implicated that the online instructional video watching made the patients worried, which might come from a better insight of coronary artery disease obtained from the video content. For the same reason, the intervention group had significantly higher faith subscale scores before discharge and at the first OPD return visit.

The results of the 90-day postdischarge follow-up showed a higher total readmission rate in the control group, especially admissions for heart failure decompensation and other comorbidities. This may be due to the instructional online video containing shared information about CAD prevention with regards to heart failure and other vascular comorbidities such as diabetes and peripheral artery disease. On the other hand, the older average age in the control group may also have contributed to the admission rate for heart failure and comorbidities.

### 4.2. Barriers in the e-Health Implementation

The barriers in implementing e-health included technology disconnect and lack of the holistic approach [26]. Previous studies have shown that group patient education or education using multimedia such as a videotape or DVD before CC can improve spiritual well-being and satisfaction in patients scheduled to receive CC [27–29]. The biggest difference in this study is the application of Internet-based social media as the main tool for patient education, which overcomes the limits on time and space in addition to provision of standardized and comprehensive contents. The advantages of this tool include good accessibility, cost, and time efficiencies, and that the content can be accessed using a variety of devices. The patients can then select to watch the corresponding video scenes at anytime and anywhere to remind themselves about the periprocedural precautions or knowledge of CAD prevention in which they are interested in. Repeatedly watching the video will reinforce its contents and improve the level of self-care. This may be why there was stronger faith showed in FACIT–Sp-12 in the intervention group.

### 4.3. Prospects of e-Health in Holistic Healthcare

Holistic healthcare describes patient-centered approaches and interventions that are meant to satisfy a patient's physical, mental, emotional, and spiritual needs [30]. The primary goal for worldwide healthcare intervention is patient engagement [31], which is characterized by three dimensions: a behavior dimension (what the patient dose), a cognitive dimension (what the patient thinks and knows), and an emotional dimension (what the patient feels) [32]. The holistic approach with e-health interventions should be devoted to foster patient engagement in the future. Although the effects of the social media intervention in this study almost meet the above three dimensions for the patients with CAD, it does have room for improvement. In view of the rise of artificial intelligence (AI) in healthcare applications, AI is changing the way how healthcare is delivered, especially in personalized medicine and access to recommendations and automated treatments [33]. With the incorporation of AI and e-health, more novel patient-centered interventions other than social media application may be developed to provide more comprehensive and cost- and time-efficient accesses to satisfy the patient's need.

## 5. Limitations

The present study had several limitations. Based on a prospective quasiexperimental nonequivalent group design, selection bias of participants assigned to the control group and intervention group in different recruitment periods was inevitable in this study and might reduce the internal validity of this study [34]. In addition, many devices were able to connect to the Internet. The patients in the control group still could obtain the information about CC, PCI, or CABG by Internet search via their own mobile devices after traditional patient education in ward, which may add interference to the result of study and weaken the effect of the online institutional video watching as the measure of intervention in this study.

## 6. Conclusions

Access to the Internet is ubiquitous in most countries, and it continues to have an ever increasing impact on our daily life. The utilization of e-health/m-health efficiently provided information about CC to our patients. Our findings indicate that the more patients know, the better their spiritual well-being and the higher their satisfaction. To improve holistic healthcare in the modern era, the results of this study may provide some basis for the application of e-health to educate patients undergoing CC and potentially for other invasive procedures in other specialties.

## Figures and Tables

**Table 1:** Characteristics of the patients at baseline.

Characteristics	Intervention (*n* = 150)	Control (*n* = 150)	All cases (*n* = 300)	*p* value
Age (years)	62.85 ± 9.84	65.56 ± 10.15	64.2 ± 10.07	0.019
BMI (kg/m^2^)	27.22 ± 4.36	27.13 ± 4.77	27.18 ± 4.57	0.873
Male sex (*n*, %)	126 (84)	114 (76)	240 (80)	0.083
Smoking (*n*, %)	50 (33.3)	30 (20)	80 (26.7)	0.08

Medical history (*n*, %)				
Diabetes mellitus	72 (48)	73 (48.7)	145 (48.3)	0.908
Hyperlipidemia	74 (49.3)	66 (44)	140 (46.7)	0.355
Heart failure	16 (10.7)	24 (16)	40 (13.3)	0.174
Stroke	7 (4.7)	10 (6.7)	17 (5.7)	0.454
Peripheral artery disease	1 (0.7)	4 (2.7)	5 (1.7)	0.176
COPD	2 (1.3)	5 (3.3)	7 (2.3)	0.251

Revascularization therapy (*n*, %)				
Percutaneous coronary intervention	68 (45.3)	70 (46.7)	138 (46)	0.817
Coronary artery bypass grafting	6 (4)	2 (1.3)	8 (2.7)	0.152
Length of hospital stay (days)	3.54 ± 2.55	4.89 ± 5.07	4.22 ± 4.07	0.004

Coronary artery disease medication (*n*, %)				
Antiplatelet	139 (92.7)	139 (92.7)	278 (92.7)	0.833
ACEI/ARB	90 (60.4)	83 (55.3)	173 (57.7)	0.375
Beta-blocker	107 (73.2)	114 (76)	223 (74.6)	0.572
Mineralocorticoid receptor antagonist	9 (6)	10 (6.7)	19 (6.3)	0.824
Statin	118 (79.2)	117 (78)	235 (78.3)	0.801
Nitrate	46 (30.9)	60 (40)	106 (35.3)	0.099

BMI, body mass index; COPD, chronic obstructive pulmonary disease; ACEI, angiotensin-converting enzyme inhibitors; ARB, angiotensin-receptor blockers. Data are presented as mean ± standard deviation or frequency and percentage in parenthesis.

**Table 2:** Results of the EQ-5D and FACIT–Sp12 for the quality of holistic healthcare.

	Intervention (*n* = 150)	Control (*n* = 150)	All cases (*n* = 300)	*p* value
EQ-5D				
EQ-5D-5L (5–25)				
Before catheterization	7.19 ± 1.39	7.71 ± 2.15	7.45 ± 1.83	0.014
Before discharge	5.46 ± 0.7	6.16 ± 1.84	5.81 ± 1.43	<0.001
Postdischarge, 1^st^ OPD visit	5.23 ± 0.55	6.0 ± 1.76	5.61 ± 1.35	<0.001

EQ-VAS (0–100)				
Before catheterization	74.79 ± 9.53	73.20 ± 11.86	74 ± 10.77	0.201
Before discharge	78.76 ± 6.78	78.33 ± 8.86	77.55 ± 7.87	0.644
Postdischarge, 1^st^ OPD visit	80.75 ± 5.74	79.67 ± 8.37	79.97 ± 8.28	0.201

FACIT–Sp12 (0–48)				
Before catheterization	31.83 ± 4.17	32.79 ± 4.77	32.31 ± 4.49	0.066
Before discharge	36.63 ± 3.59	35.88 ± 4.3	36.26 ± 3.97	0.106
Postdischarge, 1^st^ OPD visit	37.61 ± 3.38	36.65 ± 4.54	37.13 ± 4.02	0.042

Mean (0–32)				
Before catheterization	22.48 ± 2.80	22.84 ± 3.37	22.66 ± 3.10	0.315
Before discharge	24.10 ± 2.47	24.63 ± 2.89	24.36 ± 2.70	0.090
Postdischarge, 1^st^ OPD visit	24.14 ± 2.40	24.92 ± 3.01	24.53 ± 2.74	0.016

Faith (0–16)				
Before catheterization	9.35 ± 1.98	9.95 ± 2.55	9.65 ± 2.30	0.025
Before discharge	12.53 ± 1.86	11.25 ± 2.15	11.90 ± 2.10	<0.001
Postdischarge, 1^st^ OPD visit	13.46 ± 1.71	11.73 ± 2.08	12.60 ± 2.09	<0.001

OPD, outpatient department; VAS, visual analogue scale; FACIT–SP, the Functional Assessment of Chronic Illness Therapy–Spiritual Well-Being Scale. Data are presented as mean ± standard deviation.

**Table 3:** Readmission during 90 days of follow-up.

Postdischarge, 90-day follow-up	Intervention (*n* = 150)	Control (*n* = 150)	All cases (*n* = 300)	*p* value
Readmission, (*n*, %)	14 (9.3)	28 (18.7)	42 (14)	0.016
Etiology of readmission				—
Expected readmission	4 (2.7)	5 (3.3)	9 (3)	
Acute coronary syndrome	3 (2)	1 (0.7)	4 (1.3)	
Heart failure	0 (0)	5 (3.3)	5 (1.7)	
Infection	2 (1.4)	5 (3.3)	7 (2.3)	
Comorbidities (PAD, CKD, and DM)	0 (0)	5 (3.3)	5 (1.7)	
Others	5 (3.4)	7 (4.7)	12 (4)	

PAD, peripheral artery disease; CKD, chronic kidney disease; DM, diabetes mellitus.

## Data Availability

All the datas are available in the text.

## Abbreviations

e-health: Electronic health
CVD: Cardiovascular disease
CAD: Coronary artery disease
CC: Cardiac catheterization
PCI: Percutaneous coronary intervention.

## Supplementary Materials

Supplementary Materials Table S1. Multivariate linear mixed model to evaluate the intervention effect on the outcome measures.
