# Supplementary material for: Role of an e-Health Intervention in Holistic Healthcare: A Quasiexperiment in Patients Undergoing Cardiac Catheterization in Taiwan
Source: J Healthc Eng. 2021 Mar 17;2021:6692952. doi: 10.1155/2021/6692952 (PMC7990527; doi:10.1155/2021/6692952)
Supplement: Supplementary Materials — Table S1. Multivariate linear mixed model to evaluate the intervention effect on the outcome measures. [file 6692952.f1.docx]

Table S1: Multivariate linear mixed model to evaluate the intervention effect on the outcome measures

|  | EQ-5D | |  | Self-perceived health status | |  | FACIT-SP12 | |
| --- | --- | --- | --- | --- | --- | --- | --- | --- |
| Parameter | *B* (95% CI) | *P* |  | *B* (95% CI) | *P* |  | *B* (95% CI) | *P* |
| **Intercept** | 7.10 (5.86 to 8.33) | <0.001 |  | 70.0 (63.5 to 76.4) | <0.001 |  | 32.7 (29.6 to 35.8) | <0.001 |
| **Treatment group** |  |  |  |  |  |  |  |  |
| Intervention *vs*. Control | -0.29 (-0.66 to -0.07) | 0.116 |  | 0.63 (-1.70 to 2.97) | 0.594 |  | -1.30 (-2.34 to -0.27) | 0.014 |
| **Time** |  |  |  |  |  |  |  |  |
| Time 2 *vs*. Time 1 | -1.56 (-1.79 to -1.24) | <0.001 |  | 4.95 (3.25 to 6.65) | <0.001 |  | 3.06 (2.36 to 3.76) | <0.001 |
| Time 3 *vs*. Time 1 | -1.67 (-1.98 to -1.37) | <0.001 |  | 6.26 (4.33 to 8.19) | <0.001 |  | 3.79 (3.04 to 4.55) | <0.001 |
| **Interaction** |  |  |  |  |  |  |  |  |
| Intervention × [Time 2 *vs*. Time 1] | -0.20 (-0.55 to 0.15) | 0.263 |  | -0.98 (-2.95 to 1.00) | 0.331 |  | 1.71 (0.79 to 2.62) | <0.001 |
| Intervention × [Time 3 *vs*. Time 1] | -0.29 (-0.67 to 0.09) | 0.135 |  | -0.28 (-2.59 to 2.02) | 0.810 |  | 1.81 (0.82 to 2.81) | <0.001 |
| **Control variables** |  |  |  |  |  |  |  |  |
| Age (years) | 0.01 (-0.01 to 0.03) | 0.217 |  | 0.04 (-0.05 to 0.12) | 0.422 |  | -0.01 (-0.05 to 0.03) | 0.794 |
| Male | -0.64 (-1.08 to -0.19) | 0.005 |  | 4.08 (1.97 to 6.20) | <0.001 |  | 1.89 (0.82 to 2.96) | 0.001 |
| Smoking | -0.13 (-0.37 to 0.11) | 0.27 |  | 0.18 (-1.64 to 2.01) | 0.843 |  | -0.33 (-1.22 to 0.56) | 0.469 |
| CABG | -0.80 (-1.32 to -0.27) | 0.008 |  | 2.15 (-4.02 to 8.32) | 0.492 |  | 0.35 (-1.43 to 2.13) | 0.693 |
| Length of hospitalization (day) | 0.07 (0.02 to 0.11) | 0.005 |  | -0.29 (-0.51 to -0.07) | 0.01 |  | -0.15 (-0.27 to -0.03) | 0.012 |
| Use of nitrates | 0.26 (-0.05 to 0.57) | 0.100 |  | -2.15 (-3.92 to -0.38) | 0.017 |  | -0.23 (-1.07 to 0.59) | 0.582 |

Time 1 = before catheterization; Time 2 = before discharge; Time 3 = post-discharge 1^st^ OPD visit;

B = regression coefficient; CI = confidence interval; FACIT–SP = The Functional Assessment of Chronic Illness Therapy–Spiritual Well-Being Scale; CABG = coronary artery bypass graft
